# Breakfast Practices in Malaysia, Nutrient Intake and Diet Quality: A Study Based on the Malaysian Food Barometer

**DOI:** 10.3390/nu15092197

**Published:** 2023-05-05

**Authors:** Elise Mognard, Theresia Pratiwi Elingsetyo Sanubari, Yasmine Alem, Jan Li Yuen, Neethianhantan Ari Ragavan, Mohd Noor Ismail, Jean-Pierre Poulain

**Affiliations:** 1 Chair “Food Studies: Food, Cultures & Health”, Taylor’s University, Subang Jaya 47500, Malaysia; 2 Centre for Asian Modernisation Studies (CAMS), Taylor’s University, Subang Jaya 47500, Malaysia; 3 Faculty of Social Sciences and Leisure Management, Taylor’s University, Subang Jaya 47500, Malaysia; 4 Centre d’Études et de Recherche: Travail, Organisation, Pouvoir (CERTOP) UMR CNRS 5044, Université de Toulouse, CEDEX 9, 31058 Toulouse, France; 5 Program Studi Gizi, Fakultas Kedokteran dan Ilmu Kesehatan, Universitas Kristen Satya Wacana, Salatiga 50711, Jawa Tengah, Indonesia; 6 Centre for Community Health Studies (ReaCH), Faculty of Health Sciences, Universiti Kebangsaan Malaysia, Kuala Lumpur 50300, Malaysia

**Keywords:** breakfast consumption, diet quality, Malaysia, nutrient intake, Nutrient Rich Food index 9.3

## Abstract

Breakfast is often referred to as the “most important meal of the day” in shaping diet quality. This study established the patterns of breakfast consumption in Malaysia and assessed its contribution to the overall quality of the diet based on the 24 h recall data from the nationally representative and cross-sectional Malaysian Food Barometer (MFB, second data collection, 2018) to assess breakfast intake among adults (*n* = 1604). Diet quality was measured using the Nutrient Rich Food index (NRF) 9.3. The nutritional profile of breakfast was compared across tertiles of NRF 9.3. Overall, 89% of Malaysians consume breakfast. Breakfast was found to provide 474 kcal on average. The Malaysian daily diet was found to be rich in fats, saturated fats, total sugars, and sodium, with breakfast making a significant contribution to the daily intakes of these nutrients. Intakes of fiber, potassium, calcium, vitamins C and D, folate, iron, zinc, and magnesium were low. Breakfast was related to the overall diet quality measured by the NRF index. This study revealed that the breakfasts consumed by Malaysian adults were found to be nutritionally unbalanced. This analysis could serve as a basis for nutrient recommendations grounded in existing social and cultural breakfast patterns.

## 1. Introduction

Since the 1970s, Malaysian multicultural society has undergone rapid industrialization and urbanization, leading to substantial transformations of lifestyles, food patterns, and health outcomes [1,2]. Breakfast is often presented as the “most important meal of the day” by nutritional science, but specific guidelines are lacking. Where available, the recommendation for the total intake at breakfast should be 20% of the recommended dietary intake [3].

The International Breakfast Research Initiative (IBRI) is a global project examining breakfast consumption and recommendations on a harmonized basis. The project has been conducted across the globe to develop regional nutrient recommendations using the available nationally representative surveys. To date, the project has been completed for two regions, namely North America and West Europe Union [3,4,5,6,7,8,9,10] and in Latin America [11,12]. This present paper addresses the breakfast consumption patterns in Malaysia in the context of the IBRI. It relies on the secondary data analysis from the Malaysian Food Barometer (MFB), a nationally representative, recurrent, socio-anthropological survey that aims to provide an empirical basis for the interdisciplinary dialogue with nutrition and public health. The MFB has been previously used to further analyze the changes in food patterns [13,14] as well as the nutrition transition [15,16] in Malaysia. Malaysia is highly diverse in ethnicity—those who are currently identified by the census are Malay, Chinese, Indian and non-Malay Bumiputera. Accordingly, an ethnocultural and social perspective on breakfast in Malaysia was developed to take into account this ethnic diversity in the recommendations and implementation of public health policies [17]. The objectives of the present study were to establish the patterns of breakfast consumption in Malaysia, and to assess the breakfast’s contribution to the overall quality of the diet. These data will serve to provide policy makers with the data necessary to make recommendations on nutrient intakes in Malaysia.

## 2. Materials and Methods

### 2.1. Data Sources and Study Sample

Data were drawn from the second data collection of the MFB completed in 2018 [18,19]. MFB is a cross-sectional socio-anthropological survey that aims to provide empirical basis for the interdisciplinary dialogue on nutrition and public health. The nationally representative sample comprises 1604 individuals aged 18 years old and above who were selected based on a semi-randomized sampling approach. The random complex multistage sample was designed to be representative in terms of geographical regions within Peninsular Malaysia (Sabah and Sarawak), degree of urbanization, age, and ethnicity. The sampling stratification methodology followed the one used for MFB1 in 2013 (*n* = 2000) that was itself in line with the Malaysian Adult Nutrition Survey (MANS, *n* = 6928) conducted by the Ministry of Health in 2002 and 2003. Of the 1660 respondents, 56 were excluded for failing to meet the criteria of a nationally representative sample. Training for interviewers was conducted to harmonize fieldwork, data collection, and input. Data were collected from March to July 2018. The protocol was approved by the Human Ethics Committee of Taylor’s University (reference HEC2017/030). Detailed information on this study’s design, protocol, and methodology has been previously published [18,19].

### 2.2. Dietary Assessment

Dietary data were obtained using a single 24 h recall, which provided detailed information on all food and beverages consumed and also the relevant preparations/recipes. In order to take variations in the day-to-day intakes between weekdays and weekends into account, data were only collected from Tuesday to Saturday. Reporting of the quantities consumed was assisted by a photographic catalog in which the food intake was quantified using of the most common portion sizes used in Malaysia [20].

Each food and recipe from the 24 h recall records was encoded and then analyzed for energy, macronutrients, and micronutrients using NutritionistPro version 8.1, which included the data of the Malaysian Food Composition Table (MyFCD), updated in 2017. The database included 44 nutrients (29 mandatory and 11 optional nutrients) and 1892 foods (including prepared foods or recipes), which were categorized into 14 food groups [21]. When not available in the MyFCD, data were borrowed from the United States Department of Agriculture (USDA) National Nutrient Database [22].

Quality control of the dietary intake data was conducted in two steps. In the first step, the foods reported by a participant their quantity were reviewed while encoding in NutritionistPro and through audit of data based on preliminary analysis of the nutrient intakes. In the second step, scatter plot was used to determine the outliers of the datasets. For implausible energy intakes defined as those below 500 kcal, participants were excluded from the dataset. After validation and checking for completeness, a total of 4 participants were excluded from this study.

### 2.3. Definition of Breakfast

There are significant challenges in defining breakfast as a meal [3,23,24], particularly in such a mixed ethnic nation. In the present study, breakfast was defined as all food or beverages consumed at the first eating occasion after an overnight fast before 10 a.m. and identified by the respondent as breakfast or equivalent in local language. Additionally, there were instances where the consumption of non-nutritionally meaningful drinks was reported as the first intake of the day. To exclude intakes of such drinks, it was agreed to exclude intakes below the commonly accepted minimum of 50 kcal [11,25,26]. This definition is in line with previous research studies of the International Breakfast Initiative [3]. Based on this definition of breakfast, 1428 respondents were identified as breakfast eaters. Selection of intakes included as breakfast is illustrated in Table 1.

### 2.4. Dietary Quality by NRF 9.3

In line with previous studies within the IBRI, nutrient intakes at breakfast were compared across tertiles of overall diet quality. The objective of this analysis is to evaluate the breakfast intakes in breakfast consumers with the best diet quality, which may inform the future development of nutrient recommendations for the breakfast meal. Diet quality was measured using the Nutrient Rich Food index 9.3 (NRF 9.3), a validated measurement tool initially developed to score nutrient density of individual foods. The present variant was used to assess the quality of the total diet by measuring nutrient intakes per reference amount [27]. The determination of the nutrient density score is calculated by the sum of the percentage daily values (DVs) of the nine qualifying nutrients (protein, dietary fiber, vitamins A, C, and E, calcium, iron, potassium, and magnesium) minus the sum of the percentage for the three disqualifying nutrients to limit (total sugars, saturated fats, and sodium). In this study, vitamin E was replaced by vitamin D.

The NRF 9.3 was calculated as follows:

(1) $$NRF\;9.3=\left(NR-LIM\right)\times 100$$

with

(2) NR=∑i=19intakeienergy x 1767DVi

and

(3) LIM=∑i=13intakeienergy x 1767MRVi−1

where intake_i_ is the intake of each nutrient *i* and DV*_i_* is the daily reference value for that specific nutrient. To prevent a high intake of one of the recommended nutrients to compensate for the low intake of other nutrients, for qualifying nutrients, each percentage of the reference daily intake was truncated at 1. For disqualifying ones, only the share in excess of the recommended amount was considered. The closer the value is to the maximum score of 900, the better the quality of the overall diet.

### 2.5. Derivation of Dietary Reference Values

In using the NRF index, the Recommended Nutrients Intakes (RNIs) for Malaysia [28] were used. These reference values are age-, sex-, and physical activity-specific. The estimated average requirements per day were computed based on the relative weights of age and sex groups amongst the Malaysian population by the RNI and are presented in Table 2. The estimated average requirements per day were then used as the basis for the computation of diet quality scores. The energy requirements were derived based on basal metabolic rate multiplied by Physical Activity Level (PAL) at 1.4. There is no nutrient information available for added sugar in MyFCD. Accordingly, total sugar intakes were used instead.

### 2.6. Statistical Analysis

All statistical analyses were performed using Statistical Package for the Social Sciences (SPSS) version 26 (IMB, New York, NY, USA). The significance of the difference between breakfast and total daily intake in the energy contribution of each macronutrient was assessed using paired samples *t*-tests. The relationship between socio-descriptive categorical variables and breakfast consumption was examined using chi-square tests. The differences in quantitative variables were examined using Analysis of Covariance (ANCOVA), controlling for confounders including energy intake at breakfast, gender, age, marital status, metropolization, and occupation, which were adjusted where appropriate. Fisher’s Least Significant Difference (LSD) post hoc test was used to examine multi-comparisons between groups. The significance level was set at *p* < 0.05.

## 3. Results

A large proportion of the respondents (89%) reported eating breakfast across demographic characteristics and body weight status. As shown in Supplementary Table S1, most of the non-eaters were females, younger adults (18 to 29 years old), single, without children, people living alone, had a higher income and educational attainment, and were metropolitan dwellers. Notably, no association was found between the consumption of breakfast and ethnicity. Subsequent analyses were conducted among breakfast eaters only.

### 3.1. Contribution of Breakfast to Daily Energy and Nutrient Analysis

The mean energy and nutrient intakes of breakfast eaters are shown in Table 3. Overall, the daily intakes were high in fat, saturated fat, and sodium. Breakfast contributed toward approximately 26% of the daily intake (474 kcal of a total of 1808 kcal). Breakfast was found to be a carbohydrate-rich meal with a higher contribution of carbohydrates to the energy intake at breakfast compared to the rest of the day (52% versus 45%, *p*-Value = 0.000). Conversely, the contributions of macronutrients to energy intake at breakfast compared to daily energy intake, were lower for protein (14% versus 17%, *p*-Value = 0.000), fat (35% versus 39%, *p*-Value = 0.000) and saturated fat (14% versus 15%, *p*-Value = 0.000).

**Figure 1:**
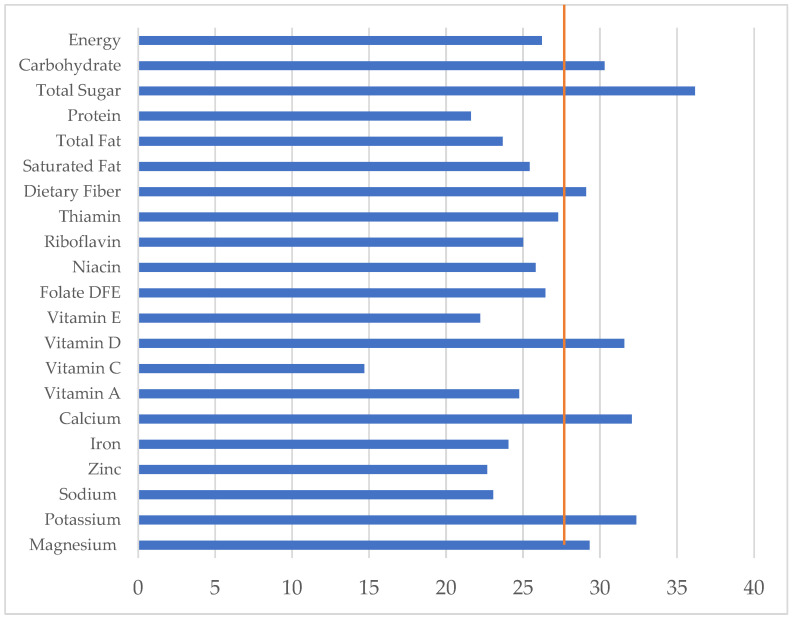
Nutrient contribution of breakfast (%) and total daily intake of Malaysian breakfast eaters (*n* = 1428) Note: The horizontal line is the percent of daily energy intake consumed at breakfast.

Figure 1 shows breakfast’s contribution to the total daily intake of energy and nutrients among Malaysian breakfast eaters. Relative to energy, breakfast contributed lower levels to daily protein, total fat, saturated fat, vitamin C, sodium, iron, and zinc intakes. Overall, breakfast contributed to more than 30% of the daily intakes of carbohydrates, vitamin D, calcium, and potassium. The total sugar intake accounted for 36% of daily consumption at breakfast.

### 3.2. Adequacy and Quality of Breakfast Nutrient Intakes 

**Figure 2:**
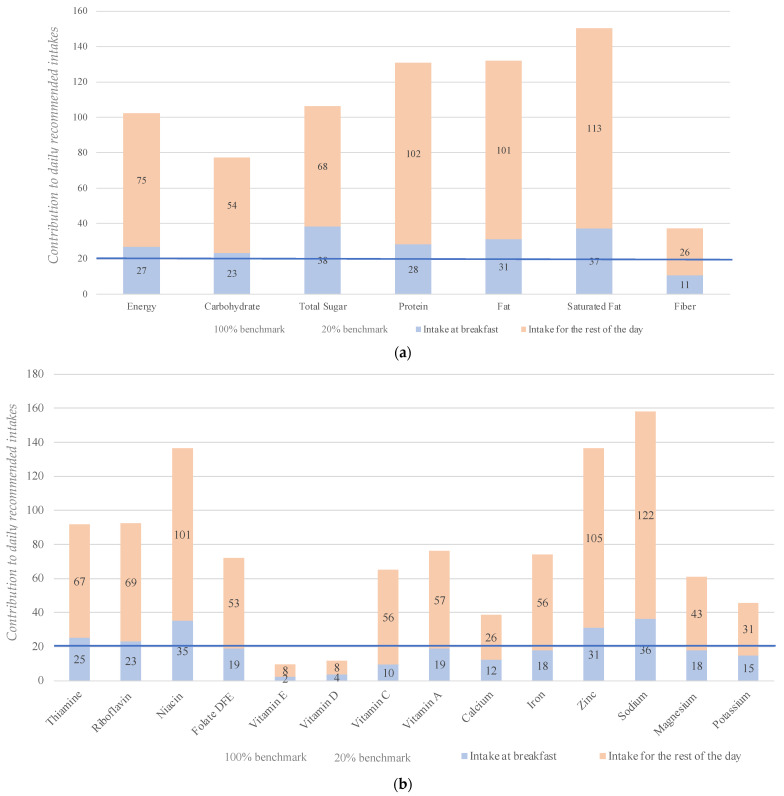
(**a**) Contribution of macronutrients within breakfast and rest of the day to daily recommended intakes for Malaysian breakfast eaters (*n* = 1428). (**b**) Contribution of micronutrients within breakfast and rest of the day compared to the daily recommended intake for Malaysian breakfast eaters (*n* = 1428). Note: 20% benchmark = 20% of the recommended intake of each nutrient (based on the RNIs), 100% benchmark = total recommendation per day (based on the RNIs).

Figure 2a,b present the contribution of breakfast and the rest of the food eaten in a day to the daily recommended intake for Malaysian breakfast eaters. Energy intake at breakfast accounted for 27% of the daily recommended intake. Relative to the commonly used target of 20% for nutrient intakes at breakfast, the present study showed that breakfast made higher contributions to the daily total sugar (~38%), proteins (~28%), fat (~31%), saturated fat (~37%), sodium (~36%), thiamine (~25%), riboflavin (~23%), niacin (~35%) and zinc (~31%) intake levels. It provided lower contributions for fiber (~11%), iron (~18%), potassium (~15%), calcium (~12%), magnesium (~18%), vitamins C (~10%), D (~4%) and E (~2%) levels. On a daily basis, the intake of sugars, total fats, saturated fats, protein, and sodium exceeded the maximum recommended intakes, while the intake levels of all micronutrients (except niacin) were significantly below the daily recommendation.

### 3.3. Breakfast Intake and Diet Quality

The breakfast eaters were divided into tertiles of daily dietary quality based on their NRF 9.3 score, and the sociodemographic and lifestyle variables were compared across the tertiles. The average NRF score for the total sample of breakfast eaters was 351. Table 4 shows the unadjusted mean nutrient intake during breakfast across different levels of the diet quality score.

After controlling for potential confounding factors (energy intake at breakfast, gender, age, marital status, metropolization, and occupation), the NRF 9.3 score was found to be significantly related to the intake of energy and all nutrients at breakfast. The mean nutrient intakes significantly differed across the NRF tertiles in all three models in the ANCOVA test. The expected gradations in nutrient intakes were observed: intakes of the NRF 9.3 nutrients increased (i.e., proteins, dietary fiber, vitamins A and C, calcium, iron, potassium, and magnesium), as did intakes of other vitamins and minerals that are not included in the NRF 9.3 score (e.g., thiamin, riboflavin, niacin, and zinc). Moreover, the intakes of two of the three NRF 9.3 nutrients to limit (total sugar and sodium) showed the expected decrease across tertiles.

An additional analysis (not shown) revealed significant differences (*p*-value = 0.046) in the NRF 9.3 between breakfast eaters (mean score = 363.5) and non-eaters (mean score = 363.5), where breakfast consumption is associated with a higher overall diet quality. The socio-demographic and socio-economic profiles of Malaysians are presented in Supplementary Table S2 across the NRF tertiles. There was a significant association between the NRF score and social position only when the lowest social position—defined as a combination of occupation and income—is associated with a lower NRF score. Notably, there was no association with any other socio-demographic variable.

## 4. Discussion

The International Breakfast Research Initiative proposes a harmonized approach across several regions and countries to the study of nutritional contribution of breakfast across the different food cultures. Previous research looking at the quality of Malaysian breakfasts has focused on school-aged children [29,30,31,32], with limited data published on adults.

### 4.1. Profile of Breakfast Non-Eaters

A high percentage (89%) of Malaysian adults consume breakfast. This finding is in agreement with results from the MANS, which found that the percentage of the population that reported eating breakfast was 89% in 2003 [33] and 94% in 2014 [34]. Together, these data indicate a stable pattern of breakfast consumption in Malaysia, with no major change in the prevalence of breakfast consumption across 20 years. Notably, a homogenous level of breakfast consumption is observed across the diverse Malaysian ethnic groups, which confirms the relative porosity and fluidity of the consumption practices of breakfast in Malaysia [17,34]. These results are in line with the previous IBRI findings in North America [4,6] and Europe [5,7,8,10], where non-consumption of breakfast was associated with a younger age. The results of the MANS study found that urbanites and younger adults were more likely to not have breakfast. Finally, socioeconomic status (SES) and its proxies (occupation, income, education attainment, and social position) are usually found to be positively associated with the omission of breakfast in other industrialized Asian countries [35,36,37,38,39].

### 4.2. Profile of Breakfast Intake

Malaysian breakfasts provided an average of 474 kcal or 26% of the daily energy intake. This is higher than the average breakfast contribution to daily energy reported globally—United States: ~17% [6], France: ~17% [5], Spain: ~17% [10], Denmark: 18–20% [7], United Kingdom: ~20% [8], Canada: ~22% [4]—and regionally, for example, ~19% in the Philippines [40] and Japan [41]. Taking into account the multiple daily eating occasions, some have suggested a range of 15–25% of the daily energy consumption as an appropriate intake at breakfast [24]. This contribution is slightly higher and is yet within the range of 20–35% of the total energy needs defined by others [42].

Relative to the commonly accepted 20% of the recommended nutrient intakes, it appears that breakfast provides an unbalanced source of nutrients in Malaysia. Contributions for total sugar, proteins, fat, saturated fats, sodium, thiamine, riboflavin, niacin, and zinc are relatively higher than the commonly used figure of 20% for the recommended intake. Conversely, the contributions are lower for fiber, potassium, calcium, and vitamins C, D, and E at breakfast. These findings illustrate, yet again, that the commonly accepted figure of 20% of all nutrients from breakfast is invalid.

Of particular interest was the finding that breakfast provided 38% of the daily recommended total sugar intake. This result reflects the high frequency of consumption of sugar and sweetened condensed milk that has been previously reported among Malaysian adults [43,44,45]. Likewise, breakfast contributed toward 36% of the daily recommended sodium intake, although it should be mentioned that the recommended daily intake of 1200 to 1500 mg is below the current recommended intakes by the World Health Organization (WHO)—i.e., 2000 mg of sodium or 5 g of salt per day. Other studies in adult Malaysians have shown that 52% exceeded the dietary sodium intake recommendation from the WHO, while sauces are a major contributor to sodium intake [46,47]. In addition, the average daily intake of saturated fat exceeded the recommended intake of 10% of the total daily energy intake, with breakfast found to be a significant source of saturated fat. This could be attributed to common breakfast dishes such as nasi lemak, fried rice, roti canai, chapati, kueh, and noodle soups. However, as previously highlighted, the consumption of these dishes is involved in the expression of the multicultural identity of Malaysia [17].

Low intakes at breakfast and thus breakfast’s contribution to the recommended intakes for micronutrients such as calcium, vitamin D, and potassium, as well as fiber were also reported in the neighboring country of the Philippines [40], while the intakes of the fat, and saturated fat at breakfast exceeded the daily recommendation by 30%. Overall, the analysis of breakfast intake profiles (as shown in Figure 1) suggests that reducing the levels of sugars and fats in breakfast dishes would be an opportunity for improvement. In this regard, after acknowledging the risks associated with excessive sugar intake for health, the Malaysian Ministry of Health has since planned and implemented several strategies and activities in order to reduce the sugar intake of its population [48]. Among those initiatives are an imposed sugar tax and the provision of nutritional advice to the Association Malaysian Gastronomy and Association of Chefs, the food operators at institutions for higher education, and the Public Facilities Training Institute. In addition, the existing National Nutrition Policy of Malaysia 2.0 [49] and the National Plan of Action for Nutrition of Malaysia 2016–2025 [50] target high intakes of fat and low intakes of fiber.

### 4.3. Contribution of Breakfast to Overall Diet Quality Score

Like all countries participating in the IBRI project, the overall diet quality was measured based on NRF 9.3 [3]. This choice was motivated by the fact that NRF 9.3 is based on the intake of nutrients rather than food groups, which makes it more appropriate in the context of the IBRI, where participating countries have diverse classifications of food groups. In the present study, the NRF 9.3 score is only associated with the position in the social stratification. The lowest position in social stratification is associated with the lowest NRF 9.3, which is consistent with past studies [6,51], highlighting the importance of including socio-cultural specificities into breakfast recommendations [17]. Besides, there is no other association between the NRF 9.3 score and the other socio-demographic characteristics. This result contrasts with previous studies where age, gender, education, occupation, income, and food security status were found to be associated with diet quality in Malaysia [52], North America [4,6], and Europe [5,7,8,10]. Locally, this might be due to the use of another diet quality measurement, such as the Malaysian Healthy Eating Index.

Breakfast consumption is associated with an overall higher diet quality, which is in line with the observations of several previous studies [53,54]. When breakfast eaters were classified into tertiles of daily dietary quality using the NRF index, the intakes at breakfast of total sugars, total fat, and sodium tended to decrease across the tertiles, while the intakes of vitamins and minerals increased. Thus, by using the nutrient intake of the top tertile as a point of reference along with the international diet guidelines, the proposed recommendations can be aligned with the established global standards and tailored to local practices. Ultimately, this approach aims to provide more effective and relevant nutrient recommendations for breakfast.

## 5. Conclusions

This is the first study to examine the nutritional quality of breakfast among Malaysian adults. One strength of the present study is that it is the first to include a nationally representative sample of the Malaysian adult population; secondly, the eating practices of the respondent were recorded in face-to-face interviews, allowing for self-definition of breakfast. The present study has provided a wide range of data on nutrient intakes at breakfast and the contribution of such intakes to the total daily diet, all of which can be used in public health nutrition programs within Malaysia.

## Figures and Tables

**Table 1:** Decision tree for the selection of breakfast eaters.

Scenario	Number of Respondents	Intake Included as Breakfast
Only one eating occasion before 10 a.m. reported and named as breakfast	1308	All food or beverages consumed at the first eating occasion before 10 a.m., provided they were more than 50 kcal
Two eating occasions before 10 a.m. but only one named as breakfast by the respondent	94	Nutrient intakes only for the eating occasion named as breakfast were included
Two eating occasions before 10 a.m. named as breakfast	22	Nutrient intakes for both eating occasions were combined and included
Two eating occasions before 10 a.m., neither named as breakfast	4	The eating occasion with the highest energy intake was chosen as breakfast
Total breakfast eaters	1428	

**Table 2:** Estimated average daily requirements used as reference values.

	Reference Values
Energy (kcal)—PAL 1.4	1767
Macronutrients	
Carbohydrate (g)	265.0
Total Sugar (g)	44.2
Protein (g)	56.6
Fat (g)	58.9
Saturated Fat (g)	19.6
Dietary Fiber (g)	29.8
Vitamins and Minerals	
Thiamine (mg)	1.2
Riboflavin (mg)	1.3
Niacin (mg)	15.9
Folate DFE (μg)	424.7
Vitamin E (mg)	9.3
Vitamin D (μg)	16.1
Vitamin C (mg)	70.0
Vitamin A RE (mg)	600.0
Calcium (mg)	1080.9
Iron (mg)	21.3
Zinc (mg)	5.5
Sodium (mg)	1498.5
Potassium (mg)	4700.0
Magnesium (mg)	369.0

Note: Calculations are based on the Recommended Nutrients Intakes (RNI) for Malaysia [29].

**Table 3:** Intake of energy and nutrients at breakfast and total daily consumption of Malaysian breakfast eaters (*n* = 1428).

	Breakfast	Daily Intake
Energy (kcal)	474 ± 302	1808 ± 628
*Macronutrient*		
Carbohydrate (g)	62.0 ± 38.4	204.7 ± 76.0
Carbohydrate (E%)	52.3 ± 16.3%	45.3 ± 12.3%
Total Sugar (g)	17.0 ± 13.2	47.0 ± 28.5
Total Sugar (E%)	14.3 ± 17.8%	10.4 ± 5.6%
Protein (g)	16.0 ± 12.4	74.0 ± 30.4
Protein (E%)	13.5 ± 6.2%	16.5 ± 4.6%
Fat (g)	18.4 ± 16.0	77.7 ± 36.3
Fat (E%)	34.9 ± 14.1%	38.5 ± 12.1%
Saturated Fat (g)	7.5 ± 7.2	29.5 ± 14.4
Saturated Fat (E%)	14.2 ± 7.0%	14.7 ± 6.0%
Dietary Fiber (g)	3.2 ± 3.5	11.0 ± 6.2
*Vitamins and Minerals*		
Thiamine (mg)	0.3 ± 0.2	1.1 ± 1.1
Riboflavin (mg)	0.3 ± 0.3	1.2 ± 0.5
Niacin (mg)	5.6 ± 4.6	21.7 ± 9.9
Folate DFE (μg)	80.9 ± 84.7	305.9 ± 165.3
Vitamin E (mg)	0.2 ± 0.5	0.9 ± 1.2
Vitamin D (μg)	0.6 ± 1.0	1.9 ± 2.4
Vitamin C (mg)	6.7 ± 15.7	45.6 ± 45.3
Vitamin A RE (mg)	113.0 ±116.7	456.6 ± 277.0
Calcium (mg)	134.3 ± 106.0	418.9 ± 267.0
Iron (mg)	3.8 ± 3.6	15.8 ± 18.0
Zinc (mg)	1.7 ± 1.5	7.5 ± 3.5
Sodium (mg)	545.8 ± 784.3	2366.9 ± 1094.0
Potassium (mg)	692.4 ± 393.1	2143.6 ± 869.2
Magnesium (mg)	65.8 ± 44.8	224.4 ± 101.9

**Table 4:** Mean energy and nutrient intake at breakfast in Malaysian breakfast eaters by NRF 9.3 score tertiles (*n* = 1428).

	All	NRF Tertiles (*n* = 1428)			*p*-Value ^1^	*p*-Value ^2^	*p*-Value ^3^
		T1	T2	T3			
NRF 9.3 Score	363.5 ± 120.8	232.1 ± 69.8	360.5 ± 29.1	492.4 ± 64.8	0.000 *	0.000 *	0.000 *
Energy (kcal)	474 ± 302	454 ± 266	489 ± 301	477 ± 333	0.200	-	-
*Macronutrients*							
Carbohydrate (g)	61.9 ± 38.4	60.4 ± 32.1	63.2 ± 38.7	62.1 ± 43.0	0.523	0.000 *	0.000 *
Total Sugar (g)	17.9 ± 13.0	21.5 ± 15.0	17.0 ±12.2	15.1 ± 10.6	0.000 *	0.000 *	0.000 *
Protein (g)	16.0 ± 12.4	13.6 ± 10.1	16.7 ± 13.0	17.3 ± 13.6	0.000 *	0.000 *	0.000 *
Fat (g)	18.4 ± 15.7	18.0 ± 16.0	19.1 ± 15.4	18.2 ± 16.0	0.001 *	0.000 *	0.000 *
Saturated Fat (g)	7.5 ± 7.3	7.3 ± 7.3	7.6 ± 7.2	7.5 ± 7.3	0.715	0.000 *	0.000 *
Dietary Fiber (g)	3.2 ± 3.5	2.5 ± 2.3	3.3 ± 3.4	3.7 ± 4.1	0.000 *	0.000 *	0.000 *
*Vitamins and Minerals*							
Thiamin (mg)	0.3 ± 0.2	0.3 ± 0.2	0.3 ± 0.2	0.4 ± 0.6	0.001 *	0.000 *	0.000 *
Riboflavin (mg)	0.3 ± 0.3	0.3 ± 0.3	0.3 ± 0.2	0.4 ± 0.3	0.001 *	0.000 *	0.000 *
Niacin (mg)	5.6 ± 4.6	4.7± 4.0	5.7 ± 4.7	6.3 ± 5.1	0.000 *	0.000 *	0.000 *
Folate DFE (μg)	81.0 ± 84.7	66.0 ± 59.5	81.0 ± 82.5	94.2 ± 102.5	0.000 *	0.000 *	0.000 *
Vitamin E (mg)	0.2 ± 0.5	0.1 ± 0.3	0.2 ± 0.4	0.2 ± 0.7	0.832	0.000 *	0.000 *
Vitamin D (μg)	0.6 ± 1.1	0.4 ± 0.7	0.5 ± 1.0	0.8 ± 1.4	0.000 *	0.000 *	0.000 *
Vitamin C (mg)	6.7 ± 15.8	6.0 ± 15.0	6.3 ± 12.4	7.8 ± 19.1	0.169	0.000 *	0.000 *
Vitamin A_RE (mg)	113.1 ± 117.0	88.7 ± 85.3	112.5 ± 110.1	137.0 ± 141.6	0.000 *	0.000 *	0.000 *
Calcium (mg)	134.3 ± 106.0	119.8 ± 79.8	136.2 ± 107.0	149.6 ± 135.8	0.000 *	0.000 *	0.000 *
Iron (mg)	3.8 ± 3.6	3.1 ± 2.3	3.8 ± 3.4	5.1 ± 11.2	0.000 *	0.000 *	0.000 *
Zinc (mg)	1.7 ± 1.5	1.4 ± 1.1	1.8 ± 1.6	1.9 ±1.7	0.000 *	0.000 *	0.000 *
Sodium (mg)	545.8 ± 479.0	622.6 ± 569.5	571.8 ± 447.3	446.6 ± 390.0	0.000 *	0.000 *	0.000 *
Potassium (mg)	592.4 ± 393.1	521.7 ± 311.1	601.0 ± 386.8	653.8 ± 461.9	0.000 *	0.000 *	0.000 *
Magnesium (mg)	65.8 ± 44.8	56.0 ± 33.0	66.5 ± 43.9	74.5 ± 52.8	0.000 *	0.000 *	0.000 *

Note: ^1^ Unadjusted *p*-values; ^2^ Adjusted *p*-values for energy intake at breakfast; ^3^ Adjusted *p*-values for factors such as energy intake at breakfast, gender, age, marital status, metropolization, and occupation; * significant results.

## Data Availability

Public availability of the dataset from the 2018 Malaysian Food Barometer by the Chair of Food Studies “Food Cultures and Health” based on Open Science philosophy is in progress. Raw data sets are available on request from the corresponding author.

## Supplementary Materials

The following supporting information can be downloaded at https://www.mdpi.com/article/10.3390/nu15092197/s1, Table S1: Socio-demographic characteristics and body weight status of respondents and association with breakfast practices; Table S2: Socio-demographic characteristics and body weight status distribution of NRF 9.3 scores by tertiles.
