# Supplementary material for: Breakfast Practices in Malaysia, Nutrient Intake and Diet Quality: A Study Based on the Malaysian Food Barometer
Source: Nutrients. 2023 May 5;15(9):2197. doi: 10.3390/nu15092197 (PMC10180565; doi:10.3390/nu15092197)
Supplement: Supplementary file 1 [file nutrients-15-02197-s001.zip › nutrients-2372196-supplementary.pdf]

Table S1. Socio-Demographic Characteristics and Body Weight Status of Respondents and Association with Breakfast Practices

	Malaysian Food Barometer 2		Breakfast Non-Consumer	Breakfast Consumer
	N	%	N (%)	N (%)
**All**	1604	100	176 (11.0)	1428 (89.0)
**Gender**				
Male	729	45.4	65 (8.9)	664 (91.1)
Female	875	54.6	111 (12.7)	764 (87.3)
P-Value[1]	***0.016****			
**Age groups (years)**				
18-29	616	38.4	116 (18.8)	500 (81.2)
30-59	873	54.4	57 (6.5)	816 (93.5)
60 and above	115	7.2	3 (2.6)	112 (97.4)
P-Value[1]	***0.000****			
**Ethnicity**				
Non-Malay Bumiputra	207	12.9	27 (13.0)	180 (87.0)
Malay	888	55.4	91 (10.2)	797 (89.9)
Indian	119	7.4	20 (16.8)	99 (83.2)
Chinese	390	24.3	38 (11.0)	352 (90.3)
P-Value[1]	***0.105***			
**Marital status**				
Single/Widow/Divorced/ Separated	815	50.8	122 (15.0)	693 (85.0)
Married/ living together	789	49.2	54 (6.8)	735 (93.5)
P-Value[1]	***0.000****			
**Number of children**				
No Children	862	53.7	133 (15.4)	729 (84.6)
1-2 Children	385	24.0	26 (6.8)	359 (93.2)
3 or more Children	357	22.3	17 (4.8)	340 (95.2)
P-Value[1]	***0.000****			
**Number of family members**				
1 Person	42	2.6	10 (23.8)	32 (76.4)
2 Persons	129	8.0	21 (16.3)	108 (83.7)
3-5 Persons	1024	63.8	97 (9.5)	927 (90.2)
6 Persons and above	409	25.5	48 (11.7)	361 (88.3)
P-Value[1]	***0.004****			
**Urbanization**				
Urban	1461	91.1	159 (10.9)	1302 (89.1)
Rural	143	8.9	17 (11.9)	126 (88.1)
P-Value[1]	***0.714***			
**Metropolization**				
Metropolitan city	656	40.9	86 (13.1)	570 (86.9)
Rural peninsular	631	39.3	51 (8.1)	580 (91.1)
Sabah Sarawak	317	19.8	39 (12.3)	278 (87.7)
P-Value[1]	***0.011****			
**Highest level of education**				
Primary or lower	124	7.7	2 (1.6)	122 (98.4)
Lower secondary school	260	16.2	17 (6.5)	243 (93.5)
Upper secondary school	714	44.5	78 (10.9)	636 (89.1)

	Malaysian Food Barometer 2		Breakfast Non-Consumer	Breakfast Consumer
	N	%	N (%)	N (%)
College / University	506	31.5	79 (15.6)	427 (84.4)
P-Value[1]	***0.000****			
**Occupation**				
Professional	255	15.9	32 (12.5)	223 (87.5)
Blue Collar	367	22.9	33 (9.0)	334 (91.0)
White Collar	982	61.2	111 (11.3)	871 (88.7)
P-Value[1]	***0.327***			
**Average monthly individual income (Ringgit Malaysia)**				
100 to 699.99	357	22.3	34 (9.5)	323 (90.5)
700 to 1,332.99	700	43.6	64 (9.1)	636 (90.9)
1,333 to 1,999.99	237	14.8	27 (11.4)	210 (88.6)
2,000 and above	310	19.3	51 (16.5)	259 (83.5)
P-Value[1]	***0.005****			
**Dynamic of personal income over the past 5 years**				
Have decreased more than 10%	139	8.7	13 (9.4)	126 (90.6)
Have remained more or less stable or increased more than 10%	1465	91.3	163 (11.1)	1302 (88.9)
P-Value[1]	***0.523***			
**Social position**				
Low	357	50.1	34 (9.5)	323 (90.5)
Middle	1073	25.4	117 (10.9)	956 (89.1)
High	174	24.5	25 (14.4)	149 (85.6)
P-Value[1]	***0.243***			
**BMI groups (WHO classification)**				
Underweight	165	10.3	29 (17.6)	136 (82.4)
Normal	883	55.0	91 (10.3)	792 (89.7)
Overweight	383	23.9	38 (9.9)	345 (90.1)
Obesity	173	10.8	18 (10.4)	155 (89.6)
P-Value[1]	***0.041****			

[1] Chi-square test for association with α=0.05, *significant result

Table S2. Socio-Demographic Characteristics and Body Weight Status Distribution of NRF 9.3 Scores by Tertiles

	Malaysian Food Barometer 2	NRF Tertile 1	NRF Tertile 2	NRF Tertile 3
	N (%)	N(%)	N (%)	N (%)
**All**	1604 (100)	535 (33.3)	535 (33.3)	534 (33.3)
**Gender**				
Male	727 (45.4)	262 (36.0)	233 (32.0)	232 (31.9)
Female	875 (54.6)	272 (31.1)	301 (34.4)	302 (34.5)
P-Value[1]	***0.112***			
**Age groups (years)**				
18-29	615 (38.4)	216 (35.1)	211 (34.3)	188 (30.6)
30-59	872 (54.4)	280 (32.1)	276 (31.7)	316 (36.2)
60 and above	115 (7.2)	38 (33.0)	47 (40.9)	30 (26.1)
P-Value[1]	***0.054***			
**Ethnicity**				
Non-Malay Bumiputra	206 (12.9)	71 (34.5)	67 (32.5)	68 (33.0)
Malay	887 (55.4)	304 (34.3)	286 (32.2)	297 (33.5)
Indian	119 (7.4)	49 (41.2)	40 (33.6)	30 (25.2)
Chinese	390 (24.3)	110 (28.2)	141 (36.2)	139 (35.6)
P-Value[1]	***0.130***			
**Marital status**				
Single/Widow/divorced/ separated	814 (50.8)	283 (34.8)	272 (33.4)	259 (31.8)
Married/ living together	788 (49.2)	251 (31.9)	262 (33.2)	275 (34.9)
P-Value[1]	***0.339***			
**Number of children**				
No Children	860 (53.7)	290 (33.7)	294 (34.2)	276 (32.1)
1-2 Children	385 (24.0)	125 (32.5)	131 (34.0)	129 (33.5)
3 or more Children	357 (22.3)	119 (33.3)	109 (30.5)	129 (36.1)
P-Value[1]	***0.654***			
**Number of family members**				
1 Person	42 (2.6)	11 (26.2)	11 (26.2)	20 (47.6)
2 Persons	128 (8.0)	40 (31.3)	38 (29.7)	50 (39.1)
3-5 Persons	1023 (63.8)	346 (33.8)	349 (34.1)	328 (32.1)
6 Persons and above	409 (25.5)	137 (33.5)	136 (33.3)	138 (33.3)
P-Value[1]	***0.364***			
**Urbanization**				
Urban	1459 (91.1)	497 (34.1)	484 (33.2)	478 (32.8)
Rural	143 (8.9)	37 (25.9)	50 (35.0)	56 (39.2)
P-Value[1]	***0.114***			
**Metropolization**				
Metropolitan city	656 (40.9)	206 (31.4)	222 (33.8)	228 (34.8)
Rural peninsular	630 (39.3)	208 (33.0)	216 (34.3)	206 (32.7)
Sabah Sarawak	316 (19.8)	120 (38.0)	96 (30.4)	100 (31.6)
P-Value[1]	***0.332***			
**Highest level of education**				
Primary or lower	124 (7.7)	35 (28.2)	43 (34.7)	46 (37.1)
Lower secondary school	260 (16.2)	103 (39.6)	80 (30.8)	77 (29.6)
Upper secondary school	714 (44.5)	222 (31.1)	240 (33.6)	252 (35.3)

	Malaysian Food Barometer 2	NRF Tertile 1	NRF Tertile 2	NRF Tertile 3
	N (%)	N(%)	N (%)	N (%)
College / University	504 (31.5)	174 (34.5)	171 (33.9)	159 (31.5)
P-Value[1]	***0.179***			
**Occupation**				
Professional	253 (15.9)	74 (29.2)	97 (38.3)	82 (32.4)
Blue Collar	367 (22.9)	126 (34.3)	126 (34.3)	115 (31.3)
White Collar	982 (61.2)	334 (34.0)	311 (31.7)	337 (34.3)
P-Value[1]	***0.274***			
**Average monthly individual income (Ringgit Malaysia)**				
100 to 699.99	357 (22.3)	136 (38.1)	120 (33.6)	101 (28.3)
700 to 1,332.99	699 (43.6)	221 (31.6)	246 (35.2)	232 (33.2)
1,333 to 1,999.99	236 (14.8)	76 (32.2)	72 (30.5)	88 (37.3)
2,000 and above	310 (19.3)	101 (32.6)	96 (31.0)	113 (36.5)
P-Value[1]	***0.136***			
**Dynamic of personal income over the past 5 years**				
Have decreased more than 10%	139 (8.7)	50 (36.0)	46 (33.1)	43 (30.9)
Have remained more or less stable or increased more than 10%	1463 (91.3)	484 (33.1)	488 (33.4)	491 (33.6)
P-Value[1]	***0.747***			
**Social position**				
Low	357 (50.1)	136 (38.1)	120 (33.6)	101 (28.3)
Middle	1072 (25.4)	357 (33.3)	349 (32.6)	366 (34.1)
High	173 (24.5)	41 (23.7)	65 (37.6)	67 (38.7)
P-Value[1]	***0.012****			
**BMI groups (WHO general classification)**				
Underweight	165 (10.3)	59 (35.8)	53 (32.1)	53 (32.1)
Normal	882 (55.0)	312 (35.4)	282 (32.0)	288 (32.7)
Overweight	382 (23.9)	116 (30.4)	131 (34.3)	135 (35.3)
Obesity	173 (10.8)	47 (27.2)	68 (39.3)	58 (33.5)
P-Value[1]	***0.273***			

[1] Chi-square test for association with α=0.05, *significant result
